# Soft Stretchable Conductive Carboxymethylcellulose Hydrogels for Wearable Sensors

**DOI:** 10.3390/gels8020092

**Published:** 2022-02-04

**Authors:** Kyuha Park, Heewon Choi, Kyumin Kang, Mikyung Shin, Donghee Son

**Affiliations:** 1 Department of Electrical and Computer Engineering, Sungkyunkwan University (SKKU), Suwon 16419, Korea; telos6063@gmail.com (K.P.); chwchw97@gmail.com (H.C.); zseqqq@gmail.com (K.K.); 2 Department of Biomedical Engineering, Sungkyunkwan University (SKKU), Suwon 16419, Korea; 3 Department of Intelligent Precision Healthcare Convergence, Sungkyunkwan University (SKKU), Suwon 16419, Korea; 4 Department of Superintelligence Engineering, Sungkyunkwan University (SKKU), Suwon 16419, Korea

**Keywords:** carboxymethylcellulose, alginate, polyacrylamide, silver flake composite, conductive hydrogel, soft hydrogel, stretchable hydrogel, electromyogram

## Abstract

Hydrogels that have a capability to provide mechanical modulus matching between time-dynamic curvilinear tissues and bioelectronic devices have been considered tissue-interfacing ionic materials for stably sensing physiological signals and delivering feedback actuation in skin-inspired healthcare systems. These functionalities are totally different from those of elastomers with low ionic conductivity and higher stiffness. Despite such remarkable progress, their low conductivity remains limited in transporting electrical charges to internal or external terminals without undesired information loss, potentially leading to an unstable biotic–abiotic interfaces in the wearable electronics. Here, we report a soft stretchable conductive hydrogel composite consisting of alginate, carboxymethyl cellulose, polyacrylamide, and silver flakes. This composite was fabricated via sol–gel transition. In particular, the phase stability and low dynamic modulus rates of the conductive hydrogel were confirmed through an oscillatory rheological characterization. In addition, our conductive hydrogel showed maximal tensile strain (≈400%), a low deformations of cyclic loading (over 100 times), low resistance (≈8.4 Ω), and a high gauge factor (≈241). These stable electrical and mechanical properties allowed our composite hydrogel to fully support the operation of a light-emitting diode demonstration under mechanical deformation. Based on such durable performance, we successfully measured the electromyogram signals without electrical malfunction even in various motions.

## 1. Introduction

Soft and stretchable devices that can intimately interface with various biological tissues, such as skin, brain, heart as well as peripheral nerves have attracted huge attention in realizing the ultimate closed-loop bioelectronics capable of personalized healthcare monitoring and feedback precise treatment [1,2,3,4,5,6]. In the beginning of such devices, their stretchability with low stiffness was achieved by adopting some deformable designs such as a buckled structure in a neutral mechanical plane or rigid active cell island with wavy interconnects [7,8,9]. However, this approach remains critical due to the challenges associated with high-cost microfabrication processes and low areal density. To overcome such limitations, a materials-driven approach, “intrinsically stretchable electronics”, was newly introduced [10,11,12]. The point is that conductive stretchable composite materials can replace the wavy interconnects/electrodes fabricated through the previous structural deformable designs. The conductive stretchable composites are generally formed by mixing elastomers (e.g., polydimethylsiloxane (PDMS), styrene ethylene/butylene styrene (SEBS)) or hydrogels with conductive fillers (metal particles, carbon materials, or conjugated polymers) [13,14,15,16]. Despite the remarkable progresses of the elastomer-based conductive composites, their stiffness values that are higher than those of the living tissues may lead to chronic tissue irritation or electrical performance degradation.

To solve these issues, hydrogels with high water contents are good candidates due to their tissue-like stiffness, ionic conductivity, and even long-term biocompatibility. Most hydrogels are fabricated through chemical crosslinking or the physical entanglement of natural polymers [17,18,19,20,21,22,23,24]. From the aspect of fabrication cost, the use of natural polymers can also provide advantages for developing cheap and large-scale bioelectronics [25]. Nevertheless, the current hydrogels cannot be applied to tissue-interfacing electrodes or interconnects due to their low ionic conductivity, compared to those of metal or carbon materials [26,27,28,29]. Thus, a significant improvement of the electrical conductivity of those hydrogels is required for utilizing them as electrode and electrical circuit materials. Although much effort to enhance their conductivity has been toward embedding a versatile conductive filler in the polymeric network, such an approach result in heterogenous mechanical/electrical properties of the final products. When the conductive fillers are incorporated in pre-gel solution, their sol–gel transition via generally light-induced crosslinking can be inhibited. For homogenous gelation, the polymeric concentration as well as the amounts of initiators and monomers should be carefully optimized.

**Figure 1:**
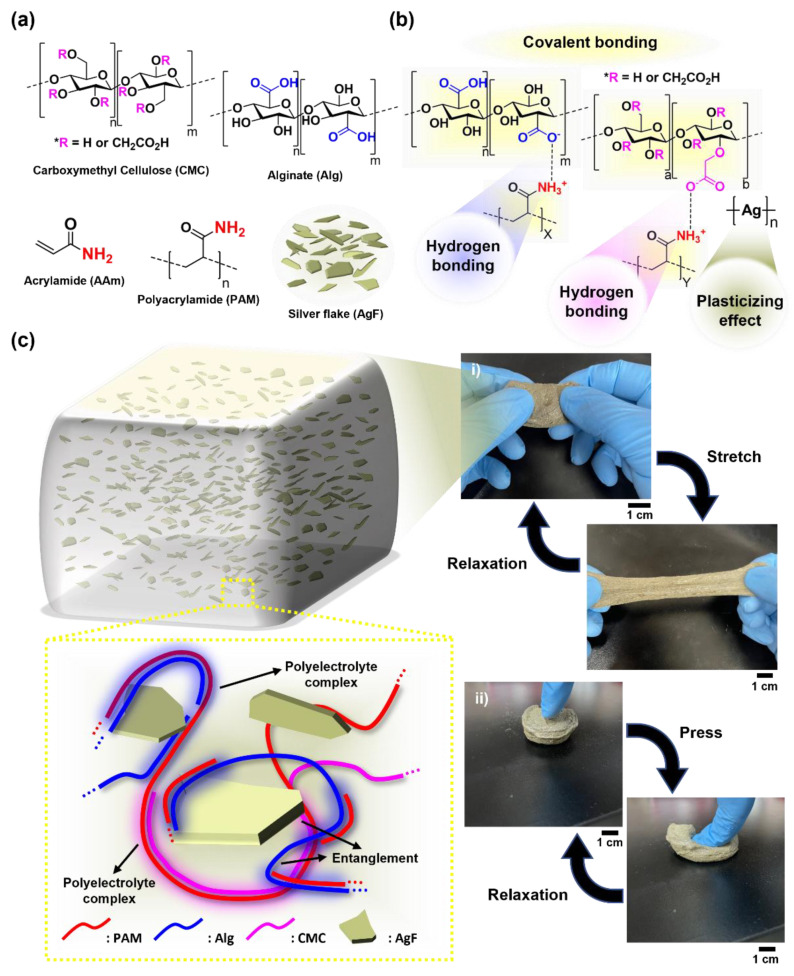
Schematic description of stretchable Alg-PAM-CMC conductive hydrogel. (**a**,**b**) Chemical structures of carboxyl cellulose (CMC), alginate (Alg), acrylamide (AAm), polyacrylamide (PAM), and silver flake (AgF) (**a**) and Alg-CMC-PAM-AgF composite-based conductive hydrogel (**b**). (**c**) Illustration describes the network structure of Alg-CMC-PAM conductive hydrogel. Macroscopic images show the flexibility (**c**, (**i**)) and stretchability (**c**, (**ii**)).

In this study, we optimized the methodology for a homogenous fabrication of conductive hydrogel composite in a way that mixes three different polymers (alginate, polyacrylamide (PAM), and carboxymethyl cellulose (CMC)) with the conductive filler (e.g., silver flake; Gaff) (Figure 1). Specifically, the CMC that is synthesized via the alkali-catalyzed reaction of cellulose partially contains hydroxy or carboxyl groups. The CMC is highly tough, owing to its triple networks (Figure 1a,b). The mechanical modulus (≈50 kPa) thanks to the conductive composite hydrogel (Alg/PAM/CMC-CHs) was effectively reduced via two issues: (i) decrease in the number of binding sites between the carboxyl and amine groups and (ii) the plasticizing effect of AgF (Figure 1c). Such low stiffness allowed our conductive hydrogel composite to be further suitable for interfacing with skin tissue. To better optimize its mechanical and electrical characteristics, the AgF concentration in the hydrogel was optimized while analyzing the rheological behaviors, mechanical strength, and stretching durability. Based on the reliable properties, we successfully demonstrated the stable operations of a light-emitting diode and electromyogram signal monitoring without electrical malfunction even in various deformations.

## 2. Materials and Methods

### 2.1. Preparation of Alg/PAM/CMC-CHs

Sodium alginate, acrylamide (AAm) (suitable for electrophoresis, ≥99%), carboxymethylcellulose sodium salt (CMC) (low viscosity), N,N-methylenebisacrylamide (MBAA) (powder, for molecular biology, suitable for electrophoresis, ≥99.5%), and ammonium persulfate (APS) (ACS reagent, ≥98.0%) were purchased from Sigma-Aldrich. Silver flakes (AgF) (DSF-500 MWZ-S) were purchased from Daejoo Electronics. Gallium indium tin eutectic, 99.99% (metal basis) (liquid metal) was purchased from Alfa Aesar.

The Alg/PAM/CMC-CH was synthesized using the sol–gel transition of Alg/PAM tough hydrogel synthesis protocols from previous studies [15,30]. First, the CMC, alginate, and AAm were dissolved in deionized water (ratio between the CMC, alginate, and AAm was 1:1:8 wt %). Then, the MBAA (0.25 × 10^−2^% of the total weight of AAm) and AgF (0%, 10%, 20%, 40%, and 80% of the total DI water wt%) were added to the mixture. The entire synthesis was performed by stirring at 400 rpm. Finally, the APS (bleaching agent) (0.03 wt % of the total weight of AAm) was added to the mixture solution, which was subsequently cast into cylindrical molds and placed in an oven at 70 °C for 5 h at approximately 40% humidity. Each completed sample was labeled: ‘Alg/PAM/CMC’ (w/o AgF = pristine), ‘AgF-1’ (10%), ‘AgF-2’ (20%), ‘AgF-4’ (40%), and ‘AgF-8’ (80%) (Table 1).

### 2.2. Rheological Characterization

The oscillation frequency sweep and continuous step strain measurements of the rheological characteristics of Alg/PAM/CMC-CHs were conducted using a TA Instruments Discovery Hybrid Rheometer 2 (TA Instruments, USA). The Alg/PAM/CMC-CHs were fabricated using bulky cylindrical molds (1.25 × 1.25 × 2 mm^3^). All rheology measurements were conducted using a parallel-plate geometry (diameter: 20 mm). Continuous step strain measurements were used to evaluate the disruption and recovery of storage (G′) and loss (G″) modulus of conductive hydrogels. These measurements were conducted on alternate strains with 0.5% and 1000% storage and loss modulus, respectively. The oscillation frequency sweep measurement was used to evaluate the change in G′ and G″ rates per frequency rate from 0.01 to 10 Hz. The gap size and axial force were maintained at 2 mm and 1 N, respectively, during all rheological characterization. The two moduli were calculated using the following equations:

*ε = ε_0_ sin (ωt), σ = σ _0_ sin (ωt + δ)* (1)

*Storage modulus (G′) = σ_0_/ε_0_ (cos δ)* (2)

*Loss modulus (G″) = σ_0_/ε_0_ (sin δ)* (3)

(*σ* is stress (Pa), *ε* is strain (mm/mm), *ω* is frequency of strain oscillation (rad(°)/s), *t* is time (s), and *δ* is phase lag between stress and strain (rad(°)/s)).

### 2.3. Universal Tensile Machine Measurement

To measure the tensile stress per stretching of the Alg/PAM/CMC-CHs controlled via different volumes of AgF, linear-shaped Alg/PAM/CMC-CHs were fabricated using narrow cylindrical molds (2.5 × 2.5 × 20 mm^3^). The experiments were performed using a universal tensile machine (UTM, INSTRON 6800), which performed measurements of two types: continuous stretching at a speed rate of 10 mm/min and cyclic stretching test (about 100 times) from 0% to 50% tensile strain at a speed rate of 1 mm/s.

### 2.4. Resistance per Stretching Measurement

To measure the resistance per stretching of the Alg/PAM/CMC-CHs, which can be used to compare the conductive performance between the different volumes of AgF and organic precursors, linear-shaped Alg/PAM/CMC-CHs were fabricated using narrow cylindrical molds (similar to the tensile strength measurement samples). The experiments were performed using a probe station (MST 5500 B, MSTECH Inc., Hwaseong, Korea); an LCR meter basic support program (4284A, Agilent Technology Inc., Santa Clara, CA, USA) with 1 kHz frequency, 0.1 V DC voltage bias, and a step motor controller (SMC-100, Ecopia Corp., Anyang, Korea); and an automatic stretch-testing machine (Stretching Tester, Jaeil Optical System Corp., Incheon, Korea). Two types of experiments were performed: continuous stretching at a speed of 0.015 mm/s and cyclic stretching test (100 times) from 0% to 50% strain at a speed of 0.3 mm/s. During the measurements, a small amount of liquid metal was dripped onto the contact area of the samples for stable electrical contact between the samples and the probe station. Conductivity was calculated using the following equation [19]:

*σ = L/(ρ × A)* (4)

where *σ* is the conductivity (S/mm), *ρ* is the resistance (Ω), *A* is the cross-section of the hydrogels (mm^2^)), and *L* is the length of the hydrogel (mm).

### 2.5. Light-Emitting Diode and Electromyogram Demonstration

To assess the performance of the Alg/PAM/CMC-CH as an electrode for a wearable sensor platform, electromyogram (EMG) signal monitoring and a demonstration using an LED were performed. First, the LED demonstration proceeded via a light-emitting diodes Bulb LED Lamp, 5 mm (white color) with a basic breadboard and a Keithley 2450 source meter (Tektronix, Inc., Clackamas, OR, USA) as a power supply. Cyclic stretching was performed from 0% to 200% tensile strain at a speed of 0.3 mm/s. Second, two samples were connected to fine wire and hook-type electrodes, and the EMG signals were monitored in real time via data acquisition equipment (DAQ, PowerLab 8/35). Transparent skin patches (3M Tegaderm, Minnesota, MN, USA) were used to establish contact between the skin and electrodes with a sampling frequency of 200 kHz.

## 3. Results and Discussion

### 3.1. Rheological Behaviors of Alg/PAM/CMC-CHs

**Figure 2:**
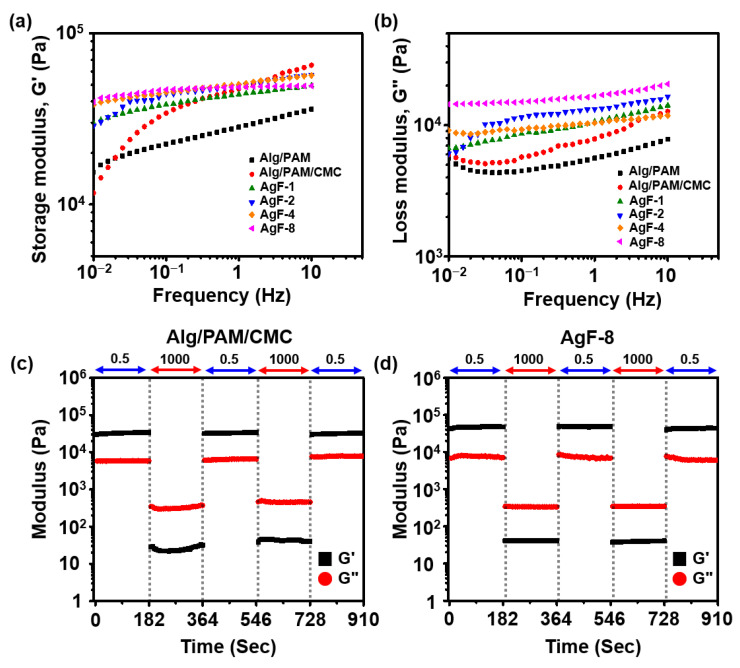
Rheological characterization of stretchable Alg- PAM-CMC conductive hydrogels containing different amounts of AgF. (**a**,**b**) Oscillation frequency sweep measurements of Alg/PAM dual-network hydrogel (Alg/PAM), Alg/PAM/CMC triple-network hydrogel (Pristine), and various Alg/PAM/CMC-CHs with different AgF ratio. (**c**,**d**) Continuous step strain measurement of Alg/PAM/CMC and Alg/PAM/CMC-CHs.

Rheological behaviors are typically evaluated through the rate distinctions and the absolute values between G′ and G″. These moduli indicate the relative energy loss with varying frequency rate, which is observable in the oscillatory behavior of parallel-plate geometry with viscoelastic material. Thus, the dynamic modulus rates from the viscoelastic material can be easily calculated (Equations (2) and (3)). The oscillation frequency sweep results show each optimum ratio between the fixed organic precursors and AgF via the gaps between G′ and G″ (Figure 2a,b). The results showed that the Alg/PAM/CMC-CHs have low dynamic modulus rates and clear rate distinctions between G′ and G″. Usually, most hydrogels made through sol–gel transitions barely satisfy both low absolute values and clear rate distinctions of G′ and G″. However, all the Alg/PAM/CMC-CHs overcome these issues. In addition, continuous step strain results showed the disruption and recovery of G′ and G″ of all samples. The results showed that Alg/PAM/CMC-CHs have a similar dynamic modulus compared to that of the pristine hydrogel (Alg/PAM/CMC) (Figure 2c,d).

### 3.2. Evaluating Tensile Stress of Alg/PAM/CMC-CHs

**Figure 3:**
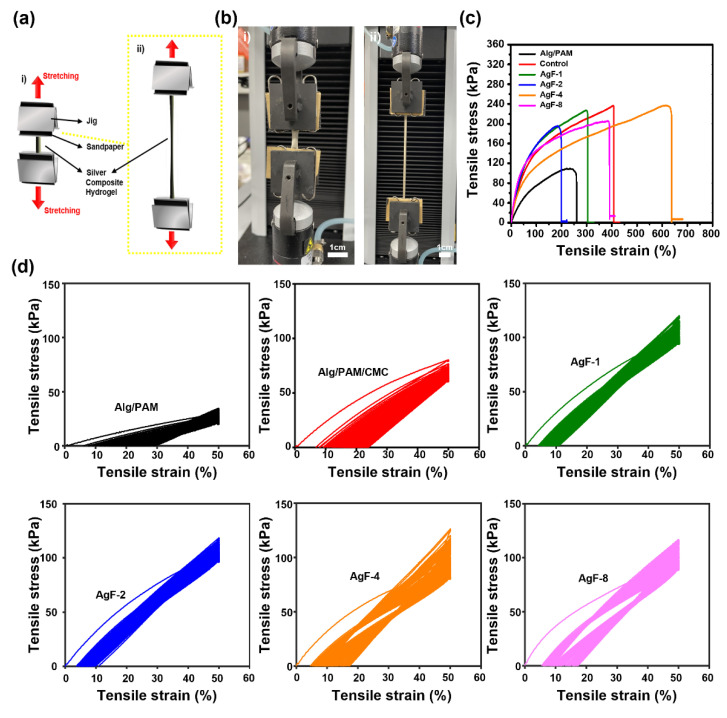
Tensile strength measurement via tensile stress per stretching. (**a**) Schematic illustration of measuring tensile strain–stress curve and (**b**) instrument (UTM) photograph. (**c**,**d**) Strain–stress curves of Alg/PAM/CMC-CH. Each measurement was proceeded via continuously strain (**c**), and cyclic strain was about 0% to 50% at a speed of 1 mm/s at 100 times (**d**).

All the candidate samples of the hydrogels for UTM measurement were protected via sandpaper for reducing deviations from mechanical mismatching with jigs (Figure 3a,b). The continuous tensile strain test was conducted to measure the maximum tensile strain rates of the hydrogel with varying toughness. The mechanical behavior of Alg/PAM could not endure more than 200% elongation, but the Alg/PAM/CMC hydrogel endured more than 400% elongation. On the other hand, compare with w/o AgF samples, in the case of Alg/PAM/CMC-CHs containing different volumes of AgF, the elongation value was significantly decreased, while the overall toughness value was decreased because of the plasticizing effect of AgF. In addition, in the case of the AgF-4 condition in which an appropriate amount of AgF was added, it looks as though the mechanical strengths were rapidly increased due to the linear energy dispersion of AgF. Furthermore, evaluating the mechanical performance of samples as a conductor of stretchable electronics, the results of AgF-4 and AgF-8 are excellent, but other Alg/PAM/CMC-CHs quickly reached the mechanical fatigue point and were thus unreasonable to use as a conductor of stretchable electronics (Figure 3c). The mechanical behaviors of cyclic stretching showed that all of the Alg/PAM/CMC-CHs could endure cyclic strains more than 100 times (Figure 3d).

### 3.3. Electrical Performance per Tensile Strain of Alg/PAM/CMC-CHs

**Figure 4:**
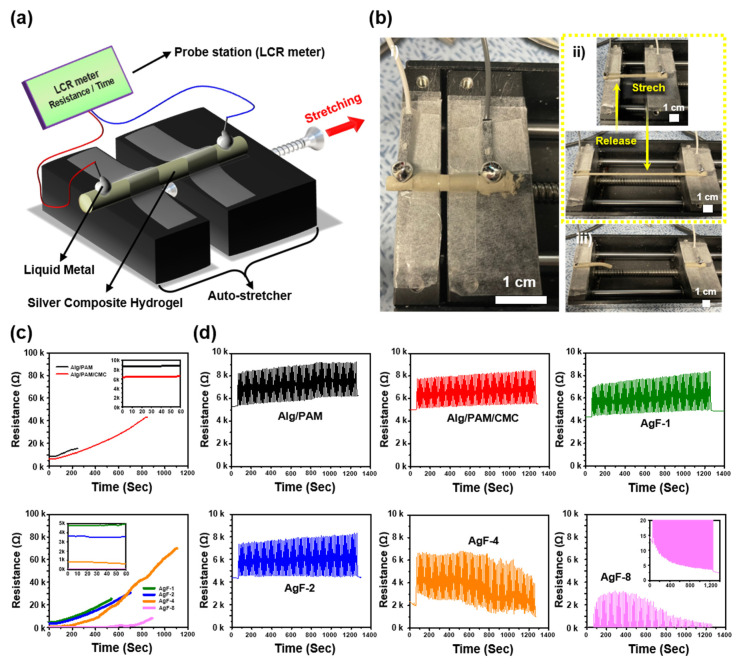
Measurements of resistance per tensile strain via different Alg/PAM/CMC-CHs. (**a**) Schematic illustration of resistance per strain test with automatic stretch-testing machine and LCR meter and (**b**) photographs showing pristine (i), stretched/released (ii), and mechanically broken Alg/PAM/CMC-CHs. (**c**) Continuous tensile strain of the samples at a speed of 0.015 mm/s. (**d**) Cyclic stretching test from 0% to 100% repetitively for strains at a speed of 0.3 mm/s at 100 times.

To analyze the varying resistance per tensile strain of the Alg/PAM/CMC-CHs, the method employed in a previous report was followed (Figure 4a,b) [19]. The results show that initially, the resistance of the pristine samples decreased exponentially when the amount of AgF in the samples increased. Similarly, varying resistance rates per tensile strain were also affected by the ratio of AgF (Figure 4c). In addition, the results of the calculated gauge factor (GF) showed that AgF-4 and AgF-8 were excellent candidates (maximum GF = AgF-4: 20, AgF-8: 241) for electrodes of the strain sensor. In contrast, the AgF-8 (8.4 Ω to 8.1 kΩ per 0% to 391% strain) will be the best for use as an integrated circuit for fabricating wearable sensor devices via 3D printing. The cyclic stretching test (0% to 100% strain repeated 100 times) showed the denaturation of resistance (Figure 4d). The results showed that AgF-4 and AgF-8 quickly denatured the strain resistance, during which significant resistance noise was detected. The results in this section suggest appropriate electrical demonstrations for each of the different Alg/PAM/CMC-CHs.

### 3.4. Electrical Performance Demonstration of Alg/PAM/CMC-CHs

**Figure 5:**
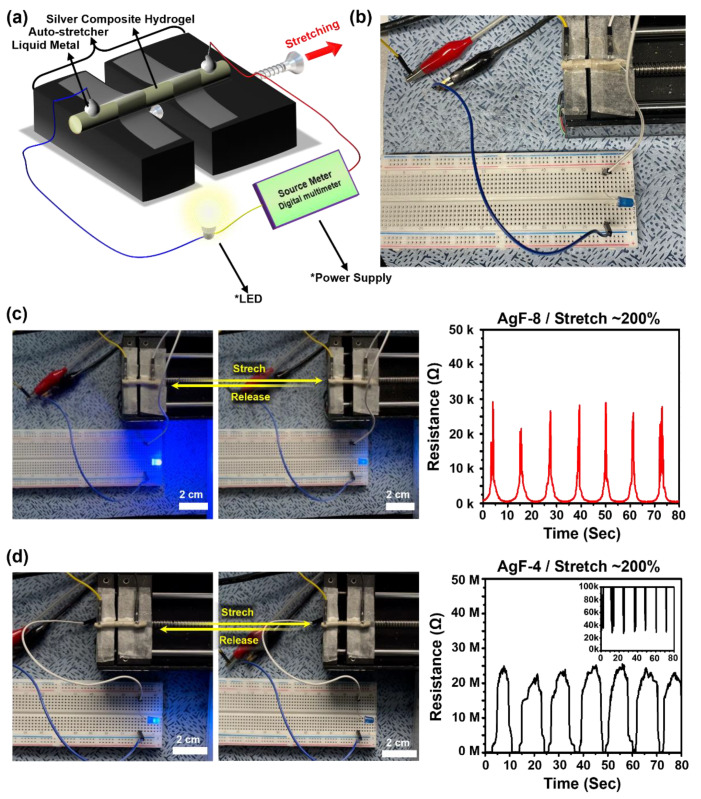
LED demonstration of Alg/PAM/CMC-CHs (AgF-8 and AgF-4) as a strain sensor electrode. Schematic illustration of LED demonstration (**a**,**b**) photograph. (**c**,**d**) Resistance per repeatedly stretch (≈200%) curve and macroscopic images of stretched and released Alg/PAM/CMC-CHs.

**Figure 6:**
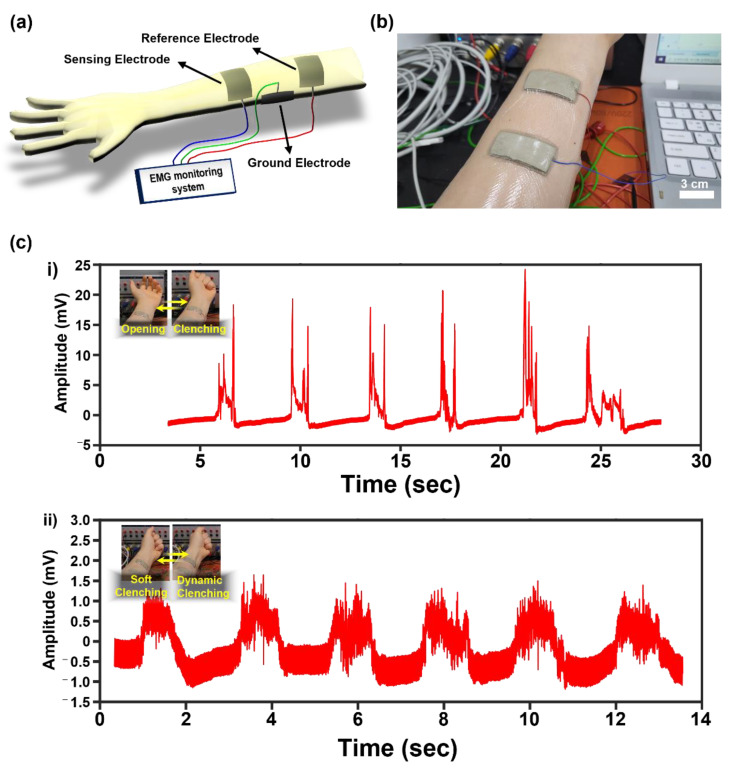
Schematic description of real-time EMG signal monitoring with Alg/PAM/CMC-CH (AgF-8). (**a**) Illustration describes the three-electrode (sensing, reference, and ground) EMG recording system and (**b**) photograph. (**c**) Amplitude changes in response to clenching-unfolding fist (**c**, (**i**)) and soft-dynamic repeated clenching fist (**c**, (**ii**)).

Two types of electrical performance demonstrations were performed. First, LED demonstrations were performed using the method described in our previous report (Figure 5a,b) [31]. The results show a luminous change in the LEDs between the stretch state and release state. In addition, the tensile strained state of AgF-4 and AgF-8 was optically confirmed (Figure 5c,d). Similarly, EMG demonstrations were performed using the method described in our previous report (Figure 6a,b) [31]. The patch type of the AgF-8 based electrode exhibited excellent signal transmission efficiency of EMG signals generated by the repeated clenching and opening of the human fist (Figure 6c).

## 4. Conclusions

In this study, we evaluated the Alg/PAM/CMC-CHs, which can be utilized as a conductor in soft electronics. The Alg, CMC, and PAM are capable of forming mild and stretchable hydrogels via sol–gel transition, which was classified as a triple-network-based composite with both strong and weak bonding. The UTM measurements and rheological characterization showed that the Alg/PAM/CMC-based triple-network hydrogel had lower toughness and higher stretchability than the Alg/PAM-based dual-network hydrogel. In addition, the UTM measurements of different Alg/PAM/CMC-CHs with different AgF contents (AgF-1, AgF-2, AgF-4, AgF-8) showed that AgF-4 had optimal mechanical properties (≈650%). Furthermore, the resistance per tensile strain measurement of Alg/PAM/CMC-CHs confirmed that a visible electrical resistance change occurred in AgF-8 at approximately ≈390% stretching (8.4 Ω to 8.1 kΩ for 2.5 × 2.5 × 20 mm^3^ sample stretching, GF = ~241). In contrast, AgF-1 exhibited less change in electrical resistance from 0% to ≈400% stretching (4.8 to 25.3 kΩ as the same volume). Finally, electrical performance demonstrations (LED and EMG) using different Alg/PAM/CMC-CHs as conductors were performed. The LED demonstration showed an evident luminous change while stretching the AgF-8 hydrogel, verifying its potential for use as a high-performance strain sensor electrode. The EMG demonstration showed the high resolution of recording signals via conformal contact between the epidermis and AgF-8 through low mechanical properties, verifying its potential for use as a wearable electronics electrode. The suggested conductive composite hydrogel has satisfactory low toughness and high conductive performance. We expect that the Alg/PAM/CMC-CH will be utilized to provide significant benefits in a high-performance wearable strain sensor.

## Figures and Tables

**Table 1:** The precursor compositions of different Alg-PAM-CMC conductive hydrogels.

	Di Water (g)	Alg (g)	CMC (g)	AAm (g)	AgF (g)	MBAA (g)	APS (g)
Alg/PAM	10	1	0	4	0	0.01	0.12
Alg/PAM/CMC	10	0.5	0.5	4	0	0.01	0.12
AgF-1	10	0.5	0.5	4	1	0.01	0.12
AgF-2	10	0.5	0.5	4	2	0.01	0.12
AgF-4	10	0.5	0.5	4	4	0.01	0.12
AgF-8	10	0.5	0.5	4	8	0.01	0.12

Alg = alginate; CMC = carboxymethyl cellulose; AAm = acrylamide; AgF = silver flake; MBAA = N’ methylenebisacrylamide; APS = ammonium persulfate.

## Data Availability

The data presented in this study are available in the article.
